# Self-Supported Nanoporous High-Entropy Alloy Electrodes with W-Modulated Surface Reconstruction for Alkaline Hydrogen Evolution

**DOI:** 10.3390/molecules31101603

**Published:** 2026-05-11

**Authors:** Furong Xu, Nana Yang, Yali Xu, Haorui Liu

**Affiliations:** 1 School of Mechanical Engineering, Lanzhou Petrochemical University of Vocational Technology, Lanzhou 730060, China; 2 School of Materials Engineering, Longdong University, Qingyang 745100, China; 3 Second Oil Production Plant, Changqing Oilfield, Qingyang 745100, China; 4 School of Materials Science and Engineering, Lanzhou University of Technology, Lanzhou 730050, China

**Keywords:** high-entropy alloy (HEA), hydrogen evolution reaction (HER), electrocatalysts, dealloying

## Abstract

Efficient and durable non-noble catalysts are crucial for alkaline hydrogen evolution (HER), and high-entropy alloys (HEAs) offer a promising platform due to their multicomponent synergy and tunable surface chemistry. Herein, self-supported nanoporous high-entropy alloy electrodes, Fe_35_Co_25_Ni_30_Mo_10_ and Fe_35_Co_25_Ni_30_Mo_7_W_3_, were prepared by arc melting followed by electrochemical dealloying in 1 M HCl. XRD results show that both alloys retain an FCC framework after dealloying, whereas SEM reveals that W promotes a more continuous sponge-like nanoporous structure. In 1 M KOH, dealloyed Fe_35_Co_25_Ni_30_Mo_7_W_3_ shows enhanced HER activity, requiring an overpotential of 178 mV at 10 mA cm^−2^, which is lower than that of dealloyed Fe_35_Co_25_Ni_30_Mo_10_ and the precursors. Dealloyed Fe_35_Co_25_Ni_30_Mo_7_W_3_ also exhibits faster kinetics (Tafel slope 98.5 mV dec^−1^; Rct 3.33 Ω) and a larger C_dl_ (19.2 mF cm^−2^) than dealloyed Fe_35_Co_25_Ni_30_Mo_10_. These results highlight W-enabled dealloying-induced reconstruction as an effective route to robust nanoporous HEA electrodes for alkaline HER.

## 1. Introduction

Hydrogen is widely regarded as an eco-friendly and energy-dense carbon-free energy carrier, and its large-scale production from renewable resources is essential for a sustainable energy future. Among various hydrogen-production technologies, water electrolysis enables the sustainable generation of high-purity hydrogen and has therefore attracted increasing attention [1,2,3,4]. However, the overall efficiency of water electrolysis critically depends on highly active and durable electrocatalysts, especially for alkaline systems that are attractive for practical deployment.

Electrocatalysts used in water electrolyzers have traditionally relied on noble metals such as Pt, Ru, and Pd, which suffer from limited abundance and high cost. Consequently, the development of non-noble-metal electrocatalysts has become a major research direction [5,6]. Nevertheless, many conventional non-precious catalysts are typically prepared as powders to maximize specific surface area, and such powdered systems often require polymeric binders and conductive additives to fabricate electrodes. These auxiliary components can introduce additional resistance, block active sites, and compromise long-term stability and scalability, thereby limiting their practical application [7,8]. Accordingly, self-supported electrocatalysts provide an attractive alternative by eliminating binders and conductive additives and enabling integrated electrode architectures with improved mechanical integrity and charge transport [9,10]. In particular, nanoporous metallic catalysts derived from dealloying have been widely explored because their bicontinuous pore/ligament networks offer large electrolyte-accessible surface areas, rapid mass transport pathways, and intrinsically high electrical conductivity [9,11,12].

High-entropy alloys (HEAs), consisting of multiple principal elements, have recently emerged as versatile electrocatalyst platforms owing to their compositional flexibility and multielement synergy [13,14]. By tuning elemental species and atomic ratios, the electronic structure and local coordination environment can be modulated, thereby enabling activity optimization for hydrogen evolution [15,16]. Combining the advantages of the nanoporous structure and HEAs, nanoporous HEAs are expected to deliver both outstanding mechanical robustness and enhanced electrocatalytic performance. For example, Yao et al. reported a hierarchical nanoporous Cu scaffold facilitating electron transport, where a nanoporous CuAlNiMoFe electrode exhibited superior HER activity under nonacidic conditions [17]. Wang et al. designed a hierarchical porous sandwich structure fabricated by dealloying a FeCoNiCuAl_2_ Mn HEA, achieving an overpotential of 9.7 mV at 10 mA cm^−2^ and a Tafel slope of 56.9 mV dec^−1^ in 1 M KOH [18]. Li et al. prepared a self-supported hierarchical porous HEA containing multiple transition metals via physical metallurgy and dealloying, where the bicontinuous mesoporous structure provided enlarged surface area and the synergistic electronic effects contributed to improved intrinsic reactivity [19].

Beyond the porous architecture, rational element selection plays a decisive role in dictating catalytic performance. Among transition metals, 3d elements such as Ni, Fe, and Co are most commonly employed as active components [20,21]. In addition, 4d and 5d metals generally exhibit broader electronic bandwidths than 3d metals, offering additional orbital degrees of freedom to regulate the electronic band structure [22,23]. High-valence metals such as Mo^6+^, Nb^5+^, and W^6+^ have also been reported to tune the electronic structure of metal oxides owing to their unique physicochemical properties [24,25,26]. These considerations suggest that incorporating 4d/5d metals into HEA systems may provide an effective strategy to modulate surface chemistry and electrocatalytic behavior.

Herein, we report the synthesis of two FeCoNi-based high-entropy alloys, FeCoNiMo and FeCoNiMoW, by arc melting, in which W is introduced to regulate the alloy chemistry. Subsequently, electrochemical dealloying in 1 M HCl is employed to construct self-supported nanoporous electrodes. The structural characteristics and alkaline HER performance of the dealloyed electrodes are systematically investigated, and the role of W incorporation in promoting the electrocatalytic performance is discussed based on comparative analyses of morphology, surface chemistry, and electrochemical metrics. This work provides insights into compositional design of nanoporous HEA electrodes and offers a practical route for developing efficient, self-supported HER catalysts.

## 2. Results and Discussion

### 2.1. Structure Characterization

**Figure 1:**
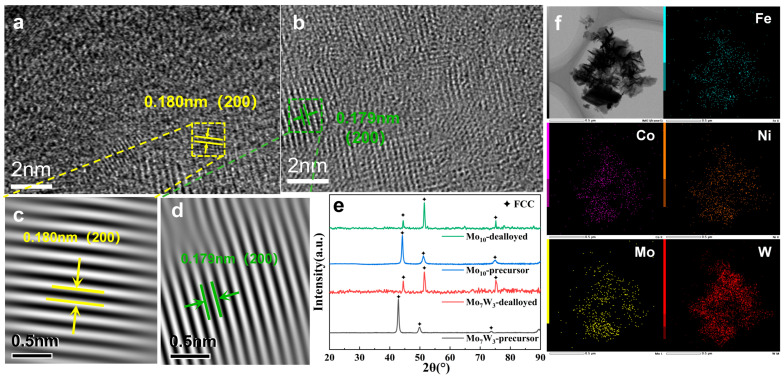
HRTEM images of (**a**) dealloyed Mo_7_W_3_, (**b**) dealloyed Mo10 and (**c**,**d**) the corresponding IFFT images. (**e**) XRD patterns of Mo_7_W_3_ and Mo_10_ before and after dealloying. (**f**) EDS mapping images of dealloyed-Mo_7_W_3_.

Figure 1e shows the XRD patterns of Fe_35_Co_25_Ni_30_Mo_10_(Mo_10_) and Fe_35_Co_25_Ni_30_Mo_7_W_3_(Mo_7_W_3_) before and after dealloying. The Mo10-precursor exhibits diffraction peaks at 44.18°, 51.12°, and 74.99°, whereas the Mo_7_W_3_-precursor shows peaks at 42.87°, 49.95°, and 73.66°, which are indexed to the FCC (111), (200), and (220) planes [23,27]. The leftward peak shift for Mo_7_W_3_-precursor is consistent with lattice expansion associated with W incorporation. After dealloying, both dealloyed Mo_7_W_3_ and dealloyed Mo_10_ largely preserve the FCC phase. Meanwhile, the relative peak intensities change, with the (111) reflection becoming weaker than the (200) reflection, which may suggest preferential dissolution and a reoriented residual framework. Notably, Mo_7_W_3_ shows a slight shift in the peaks toward higher angles after dealloying, which suggests that W addition may influence the corrosion selectivity during dealloying. In line with the XRD results, the HRTEM image (Figure 1a–d) confirms that the FCC frameworks of Mo_7_W_3_ and Mo_1_0 are both retained after dealloying. The corresponding inverse FFT (IFFT) images (Figure 1c,d) reveal interplanar distances of 0.180 nm and 0.179 nm, which correspond to the FCC (200) planes. As shown in Figure 1f, the dealloyed Mo_7_W_3_ still retains the five-element high-entropy alloy composition, with the constituent elements uniformly distributed.

**Figure 2:**
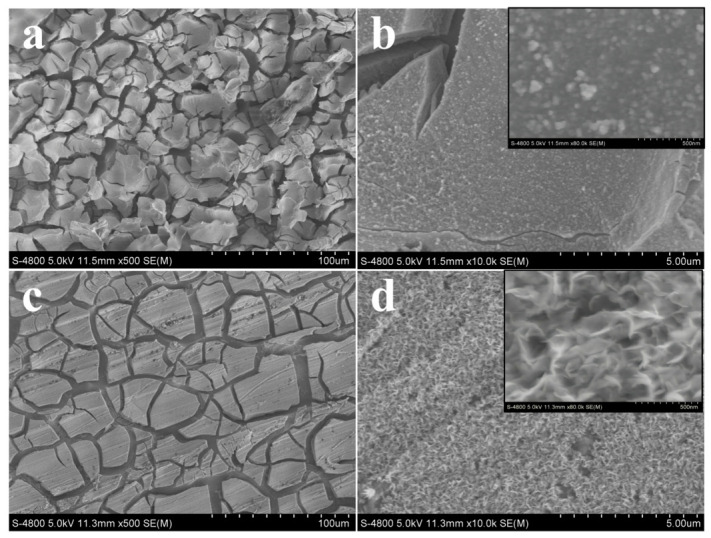
Low- and high-magnification SEM images of: (**a**,**b**) dealloyed Mo_10_ and (**c**,**d**) dealloyed Mo_7_W_3_.

After electrochemical dealloying, the two high-entropy alloys exhibit distinctly different surface morphologies. For Mo_10_, open cracks are visible at low magnification. At higher magnification, the surface remains relatively flat, with only scattered particle-like features. In contrast, Mo_7_W_3_ also exhibits cracks at low magnification, whereas higher-magnification images reveal a continuous sponge-like porous layer with an interconnected framework (Figure 2). This comparison indicates that W incorporation favors dealloying-induced surface reconstruction and promotes the formation of a more continuous nanoporous structure.

**Figure 3:**
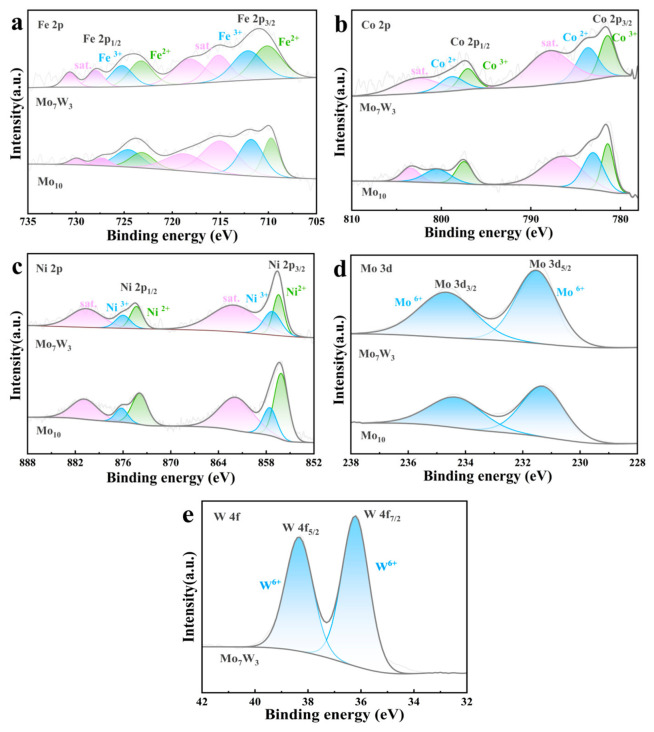
High-resolution XPS spectra of dealloyed Mo_10_ and dealloyed Mo_7_W_3_. (**a**) Fe 2p, (**b**) Co 2p, (**c**) Ni 2p, (**d**) Mo 3d, and (**e**) W 4f.

XPS analysis reveals that after dealloying, the surfaces of both alloys are predominantly composed of oxidized species, without noticeable zero-valence metals as shown in Figure 3. For dealloyed Mo_10_ and dealloyed Mo_7_W_3_, the Fe 2p, Co 2p, and Ni 2p regions can be consistently fitted with mixed-valence components (Fe^2+^/Fe^3+^, Co^2+^/Co^3+^, and Ni^2+^/Ni^3+^), accompanied by the corresponding satellite features (Figure 3a–c), indicative of the highly oxidized states of the metal elements present on the surface [28,29,30]. In addition, Mo is present exclusively as high-valence Mo species (Mo^6+^) in both alloys, as evidenced by Mo 3d_5/2_ and Mo 3d_3/2_ doublets at ~231.5 eV and ~234.5 eV (Figure 3d) [31]. Notably, W in dealloyed Mo_7_W_3_ is also in a high-valence state (W^6+^), with W 4f_7/2_ and W 4f_5/2_ peaks at 36.2 eV and 38.4 eV (Figure 3e) [32].

### 2.2. Hydrogen Evolution Performance of the Catalysts

**Figure 4:**
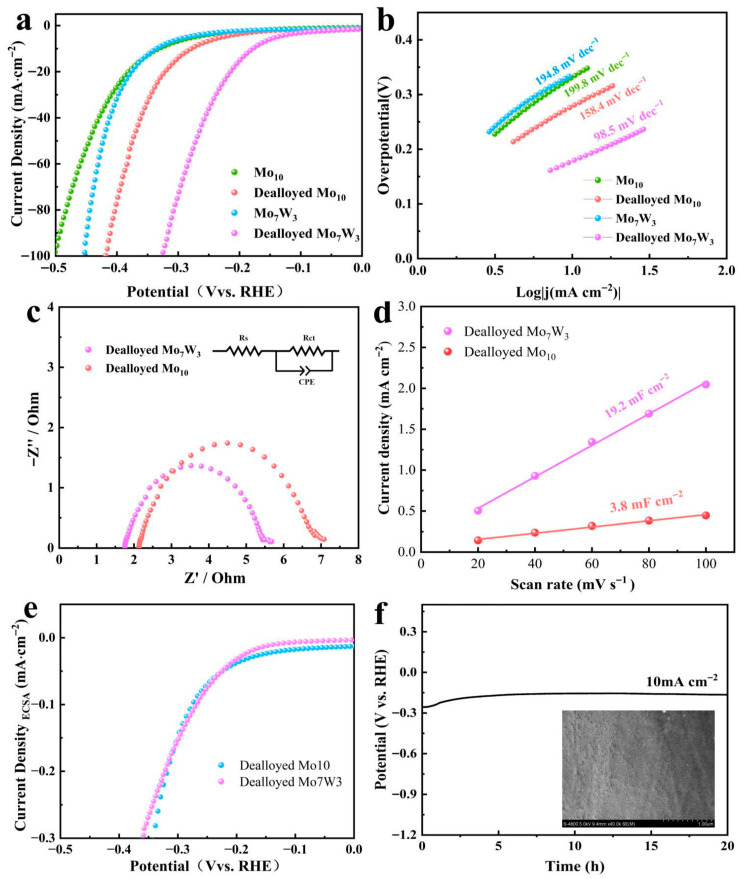
Electrochemical HER performance of prepared catalysts in 1 M KOH. (**a**) LSV curves of HER. (**b**) Tafel plots. (**c**) Nyquist plots. (**d**) C_dl_ measurements. (**e**) ECSA-normalization LSV curves of dealloyed Mo_10_ and dealloyed Mo_7_W_3_. (**f**) Chronopotentiometric curve of dealloyed Mo_7_W_3_ at 10 mA cm^−2^ and SEM image of dealloyed Mo_7_W_3_ after the stability test.

To assess the electrocatalytic performance of the prepared catalysts, the HER activity was tested in 1 M KOH electrolytes using a three-electrode system. Figure 4a compares the HER polarization curves of Mo_10_ and Mo_7_W_3_ before and after electrochemical dealloying. A clear positive shift in the LSV curves is observed after dealloying, indicating that a given cathodic current density can be achieved at less negative potentials. Quantitatively, the overpotential at 10 mA cm^−2^ decreases from 331 mV (Mo_10_) to 279 mV (dealloyed Mo_10_), and more prominently from 336 mV (Mo_7_W_3_) to 178 mV (dealloyed Mo_7_W_3_). The dealloyed Mo_7_W_3_ catalyst demonstrated the lowest overpotential among all tested samples. These results demonstrate that dealloying is effective in enhancing the HER performance for both alloys, while the W-containing alloy exhibits a much larger improvement and delivers the best HER performance after dealloying under the same testing conditions.

The HER kinetics were further assessed from the Tafel plots derived from the polarization curves. As shown in Figure 4b, the dealloyed Mo_7_W_3_ catalyst demonstrates a Tafel slope of 98.5 mV dec^−1^, which is lower than that of dealloyed Mo_10_ (158.4 mV dec^−1^), Mo_7_W_3_ (194.8 mV dec^−1^), Mo_10_ (199.8 mV dec^−1^). Importantly, after dealloying, both alloys show markedly smaller Tafel slopes. Accordingly, this trend indicates that the performance gains observed in the LSV curves are not solely attributable to higher current densities, but are also accompanied by faster apparent interfacial kinetics. Notably, dealloyed Mo_7_W_3_ exhibits a substantially lower slope than dealloyed Mo10. This difference is consistent with the SEM observation that W-containing Mo_7_W_3_ develops a more continuous, interconnected porous network, which can improve electrolyte penetration and alleviate transport limitations, thereby supporting improved HER kinetics under identical testing conditions. Moreover, the reaction kinetics was further explored by EIS analysis. As depicted in Figure 4c, the charge-transfer resistance of dealloyed Mo_7_W_3_ (3.33 Ω) is smaller than that of dealloyed Mo_10_ (4.62 Ω), indicating more efficient interfacial charge transfer. As summarized in Figure 4d, the double-layer capacitances (C_dl_) of dealloyed Mo_7_W_3_ and dealloyed Mo_10_ were determined from the slope of the linear fit of capacitive current versus scan rate, extracted from CV curves collected in a non-Faradaic potential window. The dealloyed Mo_7_W_3_ catalyst exhibits a much larger double-layer capacitance (19.2 mF cm^−2^) than dealloyed Mo_10_ (3.8 mF cm^−2^), suggesting a substantially higher electrochemically accessible surface area under identical conditions. This increased interfacial area is consistent with the more continuous porous network observed by SEM and can offer more electrolyte-accessible sites, thereby supporting higher geometric current densities. After normalization by ECSA as shown in Figure 4e, the HER polarization curves of dealloyed Mo_7_W_3_ and dealloyed Mo_10_ become very close, indicating that their area-normalized activities are comparable. This result suggests that the superior geometric HER activity of dealloyed Mo_7_W_3_ mainly originates from its much larger electrochemically accessible surface area, rather than a pronounced enhancement in intrinsic site activity. Therefore, W incorporation mainly contributes to the HER performance by promoting the formation of a more continuous nanoporous surface layer during dealloying, rather than a pronounced improvement in intrinsic site activity. The electrocatalytic stability of dealloyed Mo_7_W_3_ was confirmed under a constant current density of 10 mA cm^−2^ for 20 h by Chronopotentiometry test, as shown in Figure 4f. The dealloyed Mo_7_W_3_ catalyst exhibits stable chronopotentiometric behavior over prolonged operation, showing only a small potential fluctuation after an initial stabilization period. The absence of an obvious continuous potential increase indicates good operational durability under the tested HER conditions. Furthermore, the morphology of the dealloyed Mo_7_W_3_ catalyst preserves a nearly sponge-like porous structure even after 20 h of stability testing. In summary, the incorporation of W promotes a more favorable dealloying-induced reconstruction of Mo_7_W_3_, resulting in a continuous and interconnected porous architecture. Benefiting from the W-enabled structure, dealloyed Mo_7_W_3_ exhibits faster interfacial charge-transfer kinetics and a substantially larger electrochemically accessible surface area, thereby delivering better HER catalytic performance.

## 3. Materials and Methods

### 3.1. Preparation of Alloys

The Fe_35_Co_25_Ni_30_Mo_7_W_3_ and Fe_35_Co_25_Ni_30_Mo_10_ high-entropy alloys were synthesized by vacuum arc melting using high-purity metal elements (Fe, Co, Ni, Mo, and W, ≥99.9%). Before arc melting, the chamber was evacuated and backfilled with high-purity Ar. Then, titanium balls were melted to absorb the oxygen and other impurities in the atmosphere. Due to the high melting points of molybdenum and tungsten, which can lead to incomplete melting and compositional inhomogeneity, a binary pre-alloying strategy was employed prior to the final melting. Specifically, tungsten and molybdenum were separately pre-alloyed with nickel to form homogeneous Ni-W and Ni-Mo binary alloys, which facilitated the complete dissolution of W and Mo during subsequent melting owing to their high melting points. The pre-alloyed Ni-W and Ni-Mo ingots, along with the remaining metallic elements (Fe, Co) were then melted together. To ensure a homogeneous chemical distribution and a single-phase solid solution, the alloy ingot was subjected to five cycles of re-melting.

### 3.2. Preparation of Electrodes

The as-prepared alloy ingots were cut into 10 × 10 × 2 mm slices. The surfaces were mechanically polished to a mirror finish, followed by sequential ultrasonic cleaning in ethanol and rinsing with deionized water to remove residual contaminants. Subsequently, the samples underwent electrochemical dealloying in a conventional three-electrode system, using a graphite rod as the counter electrode and an Ag/AgCl electrode as the reference electrode. Both Fe_35_Co_25_Ni_30_Mo_7_W_3_ and Fe_35_Co_25_Ni_30_Mo_10_ were electrochemically dealloyed in 1 M HCl at −0.1 V vs. Ag/AgCl for 120 min to selectively remove components and generate a porous, catalytically active surface. After dealloying, the electrodes were thoroughly rinsed three times with ethanol and ultrapure water, and then dried under an Ar stream for subsequent electrochemical measurements.

### 3.3. Characterization

The crystal structures and phase compositions of the samples were analyzed by X-ray diffraction (XRD, D8 Advance, Bruker AXS, Karlsruhe, Germany) using Cu Kα radiation (λ = 0.15418 nm) with a step size of 0.02° and a scanning rate of 2.4°min^−1^. The surface morphology and microstructural features were observed using scanning electron microscopy (SEM, S-4800, Hitachi, Tokyo, Japan). Detailed nanostructural characterization was performed using a transmission electron microscope (TEM-EDS, JEM-2100 M, JEOL, Tokyo, Japan). The surface elemental composition and chemical states of the samples were analyzed by X-ray photoelectron spectroscopy (XPS, Axis Supra, Kratos Analytical, Manchester, UK).

### 3.4. Electrochemical Measurements

Electrochemical measurements were performed on an electrochemical workstation (Interface 1000, Gamry, Warminster, PA, USA) using a standard three-electrode configuration. The self-supported nanoporous FeCoNiMoW and FeCoNiMo alloys served directly as the working electrodes. A platinum mesh and a saturated Ag/AgCl electrode were employed as the counter electrode and reference electrode, respectively. All electrochemical tests were conducted in 1 M KOH (pH = 14) at room temperature. The measured potentials versus Ag/AgCl were converted to the reversible hydrogen electrode (RHE) using the following equation:

(1) $$E_{RHE}=E_{Ag/AgCl}+0.199+0.059\times \mathrm{pH}$$

HER activities were evaluated by linear sweep voltammetry (LSV) at a scan rate of 5 mV s^−1^ over the potential range from 0 to −0.5 V vs. RHE. The electrochemical double-layer capacitance (C_dl_) was determined from cyclic voltammetry (CV) curves recorded in a non-faradaic potential region at scan rates ranging from 20 to 100 mV·s^−1^. The Tafel slope was obtained by fitting the linear region of the polarization curves according to the Tafel equation:

(2) $$\eta =a+b\log\left|j\right|$$

where *η* is the overpotential, *b* is the Tafel slope, and *j* is the current density.

Electrochemical impedance spectroscopy (EIS) was carried out at a cathodic overpotential of −0.2 V vs. RHE over a frequency range of 100 kHz to 0.1 Hz. The diameter of the semicircle in the Nyquist plot reflects the charge-transfer resistance (R_ct_). The ECSA value was calculated by the following equation:

(3) $$\mathrm{ECSA}=\frac{C_{dl}}{C_{s}}$$

where *C_s_* is the specific capacitance of materials, which is 40 μF cm^−2^ in this paper.

## 4. Conclusions

In this work, self-supported porous FeCoNiMo and FeCoNiMoW high-entropy alloy electrodes were fabricated through high-temperature melting followed by electrochemical dealloying. XRD results indicate that both alloys retain an FCC phase before and after dealloying, indicating that the treatment mainly induces surface reconstruction rather than a bulk phase change. Notably, W incorporation likely alters the dissolution-rearrangement behavior during dealloying, enabling FeCoNiMoW to form a more continuous nanoporous structure, as supported by its much larger double-layer capacitance. Moreover, the lower Tafel slope and smaller charge-transfer resistance suggest that W may further reduce the apparent kinetic barriers for HER. This work highlights W-enabled dealloying reconstruction as a practical route to build robust nanoporous HEA electrodes and provides a simple design guideline for improving alkaline HER catalysts.

## Data Availability

Data will be made available on request.
